# Therapeutic benefits of glycerol in dry eye disease

**DOI:** 10.3389/fmed.2024.1531670

**Published:** 2025-01-15

**Authors:** Victoria Ronderos, Wendy B. Bollag

**Affiliations:** ^1^Department of Physiology, Medical College of Georgia at Augusta University, Augusta, GA, United States; ^2^Charlie Norwood VA Medical Center, Augusta, GA, United States; ^3^James and Jean Culver Vision Discovery Institute, Medical College of Georgia at Augusta University, Augusta, GA, United States

**Keywords:** cornea, dry eye disease, glycerin, glycerol, inflammation, phosphatidylglycerol

## Abstract

Dry eye disease (DED) is one of the most commonly diagnosed eye disorders, with a prevalence ranging from 5 to 50%, depending on the geographic location. DED is a multifactorial disorder of the tears and ocular surface, which results in symptoms of discomfort, visual disturbance, and tear film instability with potential damage to the ocular surface. It is also accompanied by increased osmolarity of the tear film and inflammation of the surface of the eye. Multiple therapeutic agents have been used to treat DED, with glycerol emerging as a promising component of ophthalmic preparations, due to its humectant, lubricant, demulcent, and osmoprotective properties. This review aims to evaluate the current evidence concerning the therapeutic benefits of glycerol in managing DED, focusing on its possible mechanisms of action, clinical efficacy, and potential advantages over other treatments.

## 1 Introduction

Dry eye disease (DED), also known as dry eye syndrome (DES) or keratoconjunctivitis sicca (KCS), is a common chronic and often progressive condition marked by a loss of homeostasis in the tear film, leading to symptoms such as dryness, tearing, burning, stinging, ocular fatigue, and a foreign body sensation (1, 2), as well as corneal abrasions and pain (3, 4). DED is also usually accompanied by increased osmolarity of the tear film and ocular surface inflammation (5). It is one of the world’s most commonly diagnosed eye disorders, with an estimated prevalence between 5 and 50%, depending on the geographic location (5). Because of this frequency, DED is also a burden on healthcare, estimated to cost the US system almost 4 billion dollars annually (6). Recently, there has been an increase in the incidence of DED due to multiple factors. These include, but are not limited to, contact lens use, age (increasing prevalence with age), sex (more common in females than in males), psychiatric illness, frequent computer and screen use, malnutrition (mainly omega-3 and omega-6 fatty acid and vitamin A deficiencies) (7), medications such as antihistamines and postmenopausal estrogen therapy, and refractive surgery (8). Likewise, environmental factors such as UV radiation, pollutants, and ozone can also promote the development of this disease (9). For example, DED can result from exposure to the extreme environmental conditions often found in combat areas. Indeed, a 2013 study found that almost half of male veterans 50 years or older attending an eye care clinic reported symptoms of severe DED (10). A subsequent study in Operation Iraqi Freedom and Operation Enduring Freedom veterans found that more than a quarter of these younger veterans also reported severe symptoms (11), a value similar to another study (12), suggesting the significance of DED in veterans, as well as the general population. Overall, patients with DED frequently experience a decreased quality of life with an increased risk for vision problems and blindness.

Considering the prevalence and morbidity associated with DED, there is a growing demand for effective and safe treatments for the disease. Currently, treatments for DED include artificial tears, anti-inflammatories, and punctal plugs (13). Some of these, such as topical corticosteroids, the current standard of care for corneal inflammation, can have potentially significant adverse side effects, such as ocular hypertension, glaucoma, cataracts and aggravation of infections (14, 15), indicating the importance of additional therapies to treat this condition. In recent years, glycerol has been a common ingredient in artificial tears. Glycerol is widely used in skincare, food, and pharmaceutical products. Likewise, it has been studied for its potential therapeutic effects in treating DED for its non-toxic and soothing properties. This review discusses glycerol’s possible utility as a DED therapy, including its potential mechanisms of action.

### 1.1 Characteristics and pathophysiology of dry eye disease

DED has two common subtypes: aqueous deficient dry eye (ADDE) and evaporative dry eye (EDE). These can present in patients individually or in combination. ADDE is caused by reduced tear production, usually from damage or dysfunction of the lacrimal glands. EDE develops as a result of meibomian gland dysfunction, causing an abnormality of the lipid secretion needed to regulate water evaporation and maintain a normal tear film (16). Both subtypes result in a hyperosmolar tear film, inflammation, and damage to the ocular surface. Likewise, recent studies have found that autoimmune diseases, such as Sjögren’s syndrome, rheumatoid arthritis, systemic lupus erythematosus (SLE), mixed connective tissue disease (MCTD), scleroderma, and sarcoidosis are also contributing factors to DED (17–19). Thyroid dysfunction has also been associated with DED (17).

**Figure 1:**
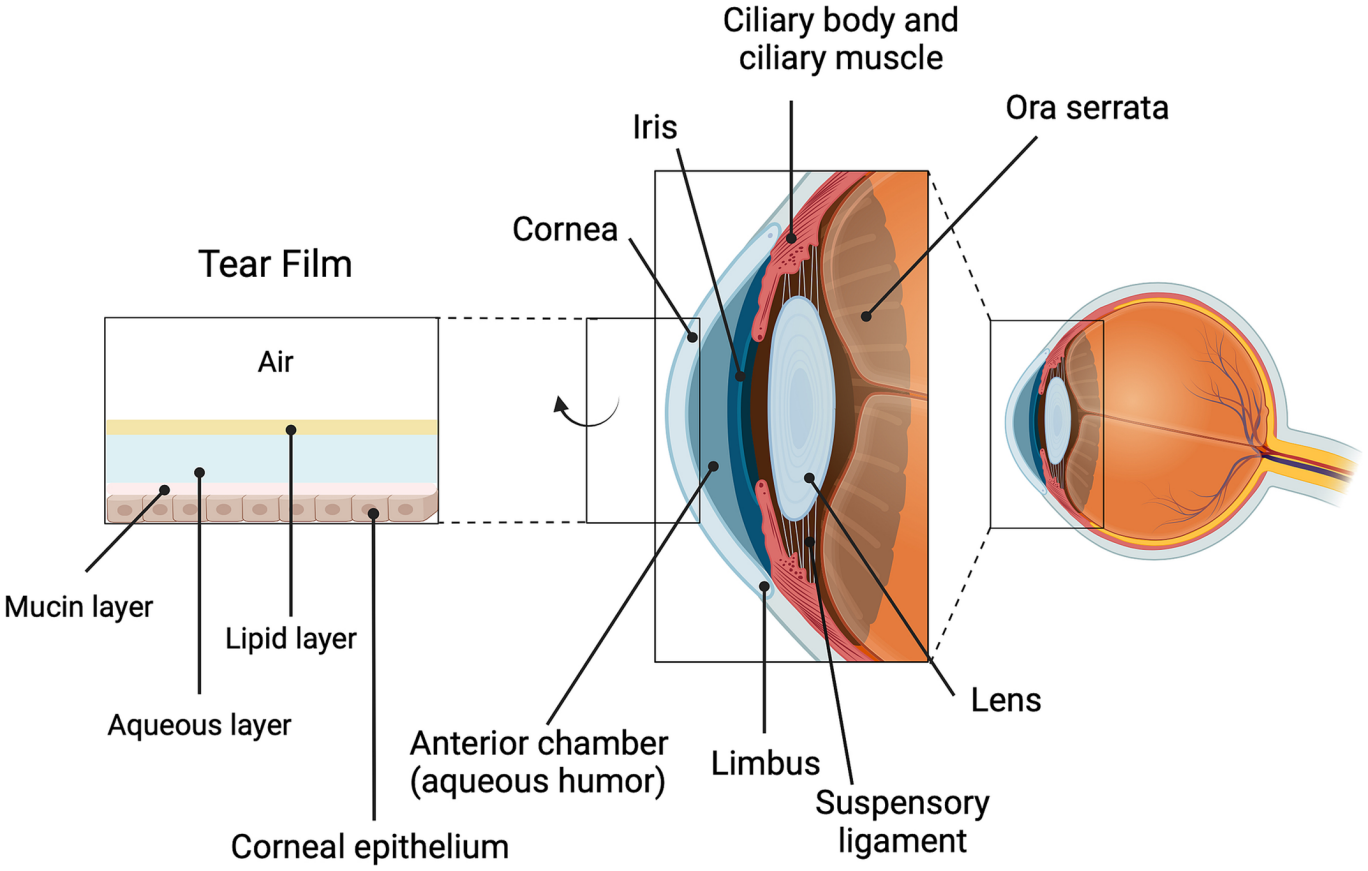
Corneal structure, including tear film components. Shown is the anterior portion of the eye with its various structures labeled, including the cornea which interfaces with the external environment. The cornea is covered by a tear film composed of a mucin layer, an aqueous layer and a lipid layer. The cornea is transparent and helps to focus the light as it enters the eye; together, the cornea and the tear film also make important contributions to the refractive capacity of the eye. Damage to the cornea, such as can occur with DED, can result in immune cell infiltration, inflammation and opacity that can compromise vision. Created using biorender.com.

DED is considered as both an acute and chronic condition. Acute DED commonly activates the innate immune system and causes an acute inflammatory response. Over time DED will activate the adaptive immune system and cause a chronic inflammatory response to become chronic DED. Chronic DED is accompanied by tear film hyperosmolarity and tear film instability, either from an increase in the evaporative loss of the tear film or a decrease in aqueous tear production. This dyshomeostasis of the tear film can then activate mitogen-activated and stress-associated protein kinases. This activation occurs in the epithelial cells of the ocular surface, and common kinases activated include p38 and c-Jun terminal kinase (JNK) (20). This increased kinase activity further stimulates the inflammatory cascade, to release chemical mediators such as vasoactive peptides and amines, pro-inflammatory cytokines and chemokines, acute phase proteins, and eicosanoids (21). Chronic inflammation can cause irreversible damage to the ocular surface, injuring the epithelial cells, the ocular nerves, and the goblet cells (20), that produce mucin, leading to corneal and conjunctival cell death via apoptosis, goblet cell loss, and poor mucus secretion (20). Likewise, DED-induced damage to the meibomian glands also further promotes the irreversible chronic inflammatory cycle (20). Given this vicious cycle, the main objectives of treating DED include restoring tear film stability, reducing inflammation, and improving patient comfort (Figure 1).

### 1.2 Properties of glycerol

Glycerol, also known as glycerin, is a three-carbon alcohol presenting as a colorless, odorless, viscous liquid commonly found in nature and in the human body (22). Glycerol is hygroscopic and a reliable humectant that attracts and retains moisture, which can help hydrate the ocular surface. The bioavailability of glycerol when applied to the eye is naturally low, considering the structural and physiological barriers of the eye. Structural barriers include the presence of hydrophilic stroma along with hydrophobic epithelium and endothelium. Physiologic barriers include lacrimal turnover, efflux transporters, blinking reflexes, and the binding of drugs to conjunctival mucins, and melatonin and tear proteins (23). Nevertheless, glycerol’s osmoprotective effects can decrease the harm caused by tear film hyperosmolarity. Accordingly, glycerol has been shown to protect cells from dehydration and environmental stress, which is particularly relevant in the context of DED, given that cellular damage from osmotic stress is common (7). Moreover, glycerol exhibits demulcent properties (4). Finally, a less often considered characteristic of glycerol is its role as a building block for synthesizing other organic molecules. In particular, glycerol is a structural component of various lipids, which comprise part of the tear film and help to reduce evaporative water loss from the ocular surface (24). For example, glycerol forms the backbone of phospholipids, neutral lipids, and the headgroup of the phospholipid, phosphatidylglycerol. All of these features of glycerol may contribute to possible beneficial effects of DED, as will be discussed.

### 1.3 Clinical evidence of glycerol’s benefit in the treatment of dry eye disease

Several clinical studies have evaluated the efficacy of glycerol-based artificial tears in treating DED, showing encouraging results in terms of improvement of signs and symptoms, as well as with regards to safety.

#### 1.3.1 Efficacy in symptom relief

Glycerol is often a component of artificial tears (25, 26), although few studies have studied the effect of isotonic glycerol alone on DED. Gensheimer et al. (27) examined the efficacy of glycerol-containing artificial tears (1%) in patients with DED. The results indicated significant improvement in objective measures of tear break-up time (TBUT), compared to Systane® Lubricant Eye Drops [with propylene glycol (0.3%) and polyethylene glycol (0.4%)] (27). TBUT is an indication of the stability of the tear film, determined as the time between the last blink and the appearance of the first dry spot in the tear film (28), with a longer TBUT time reflecting a more stable tear film. Likewise, Lidich et al. (29) examined the efficacy of glycerol-containing microemulsions as a drug delivery vehicle to the anterior chamber of the eye using benzalkonium chloride solution to mimic DED in New Zealand white rabbits. The prepared microemulsion combined Tween 80, Cremophor EL, and glycerol (2.1:1:0.2) as the surfactant phase. Glycerol was utilized to help form the microemulsion and enhance the solubility and stability of the active ingredient, docosahexaenoic acid. Indeed, the study found that this formulation significantly improved TBUT and Schirmer test scores (29). The Schirmer test score reflects the rate of tear production, with a better score indicating greater production. These findings support glycerol’s role as an effective ingredient in artificial tears for symptomatic relief in DED patients.

### 1.4 Long-term safety and tolerability

Artificial tears containing glycerol have been found to be well-tolerated with minimal adverse effects with continued use. Indeed, Kiss and Nemeth (30) explored the efficacy and tolerability of an ophthalmic formulation containing isotonic glycerol and 0.015% hyaluronic acid over a 3-month application period in patients with severe conjunctivochalasis, a disorder commonly associated with symptoms of dry eye due to the presence of conjunctival folds creating issues with the tear film (31). These authors found that adherence to the treatment plan was high, at least in part because there were no adverse events identified. In addition, the therapy was efficacious in ameliorating patients’ symptoms as well as in improving objective measures such as conjunctivochalasis, TBUT and ocular surface damage, as detected by Lissamine Green staining (26). Glycerol-based artificial tears may also be helpful for patients sensitive to preservatives, as they are often available in preservative-free formulations.

### 1.5 Comparative efficacy

Compared to other commonly used agents, such as hyaluronic acid and carboxymethylcellulose, glycerol-based tears demonstrated comparable efficacy in improving DED symptoms (3). For example, Gupta et al. (32) examined the efficacy of artificial tears containing either 1% carboxymethylcellulose (CMC) alone or 1% CMC with 0.5% glycerol. They found that patients treated with CMC plus glycerol showed more rapid improvement in TBUT, with better values measured after 1 week of treatment. Similarly, the patients treated with the combination eye drops also exhibited better Schirmer test scores after 7 days. However, at 4 weeks, comparable values were observed in both parameters in the two groups. Lievens et al. (33) obtained similar results in their study with 1% CMC with and without 0.9% glycerol with the glycerol formulation inducing earlier improvement, although the differences did not achieve statistical significance. Some studies have also suggested that glycerol’s osmoprotective properties may offer superior benefits in severe tear hyperosmolarity, positioning glycerol as a unique therapeutic option for this patient population (34).

### 1.6 Potential mechanisms of action of glycerol in dry eye disease

#### 1.6.1 Humectant effects

As a humectant, glycerol can retain moisture along the ocular surface by attracting and holding water from the surrounding environment, alleviating dryness and discomfort (34). This hydrating effect of glycerol reduces the sensation of dryness and prevents damage to the corneal epithelial cells caused by desiccation.

#### 1.6.2 Osmoprotective properties

One of the main pathologies in DED is tear film hyperosmolarity, which leads to inflammation and injury to the ocular surface. Glycerol’s osmoprotective properties help counteract this by reducing the osmotic stress on ocular surface cells (20). It stabilizes cell membranes and prevents the shrinkage of epithelial cells, thus protecting against damage from hyperosmolarity. This effect is particularly beneficial in ADDE and EDE, where the tear film’s osmolarity is often elevated.

#### 1.6.3 Viscoelasticity and tear film stabilization

Formulations that contain glycerol increase tear viscosity (25), thereby contributing to enhancement of the tear film’s stability and improvement of tear break-up time (TBUT). This stabilization of the tear film may reduce evaporation, one of the key contributors to EDE, thereby prolonging patient comfort between blinks. In addition, as noted above, glycerol can be used to synthesize lipids, which can also stabilize the tear film (24).

#### 1.6.4 Demulcent (soothing or anti-inflammatory) effects

DED is associated with chronic inflammation of the ocular surface (35, 36). Glycerol’s anti-irritant, anti-inflammatory properties, although less extensively studied, may provide an additional benefit through reducing ocular surface inflammation. Glycerol may help reduce the inflammatory response in DED in part by maintaining cellular hydration and reducing osmotic stress (20). However, there are other possible mechanisms through which glycerol might exert anti-inflammatory effects. For example, glycerol may reduce cellular oxidative stress by inhibiting the entry of hydrogen peroxide generated in response to cytokines, such as TNFα (34), into cells expressing aquaporin-3 (AQP3). AQP3 is a channel shown to allow cellular fluxes of glycerol and hydrogen peroxide, in addition to water molecules (37, 38). Choudhary et al. (39) demonstrated that the co-application of glycerol and hydrogen peroxide to AQP3-expressing skin cells (keratinocytes) reduced oxidative stress in these cells compared to those treated with hydrogen peroxide alone. This result suggests the possibility that glycerol decreases hydrogen peroxide transport into cells, perhaps by competing with hydrogen peroxide for cellular entry through AQP3.

There is another mechanism through which glycerol may provide anti-inflammatory actions: by transformation to phosphatidylglycerol. We have previously shown that in skin cells (keratinocytes), AQP3 is associated with the lipid-metabolizing enzyme phospholipase D2 (PLD2), which can use the glycerol transported by AQP3 to produce phosphatidylglycerol (40–42). In turn, we and others have shown that phosphatidylglycerol exerts anti-inflammatory effects by decreasing stimulation of the innate immune system through inhibition of toll-like receptor-2 and -4 (TLR2/4) activation. Phosphatidylglycerol can inhibit TLR2/4 activation by both microbial components, or pathogen-associated molecular patterns (PAMPs), and by endogenous proteins released by endangered or disrupted cells, called danger- or damage-associated molecular patterns (DAMPs) (43–56). In the lung, Voelker and colleagues have demonstrated that phosphatidylglycerol (primarily the species palmitoyl, oleoylphosphatidylglycerol, a component of pulmonary surfactant) inhibits inflammatory mediator production and/or inflammation induced by respiratory syncytial virus, influenza A (H1N1) and *Mycoplasma pneumoniae* (46–55). We have demonstrated that phosphatidylglycerol can also inhibit skin inflammation in an irritant application mouse model as well as a mouse model of psoriasis (45, 57). In addition, we have shown that AQP3 and PLD2 are also associated in corneal epithelial cells, as these two proteins are in skin cells (58). Furthermore, a specific phosphatidylglycerol, dioleoylphosphatidylglycerol (DOPG) accelerates corneal wound healing in wild-type mice and a mouse model of impaired corneal wound healing (AQP3 knockout mice) (58). We have hypothesized that one mechanism by which DOPG enhances corneal wound healing is by reducing (but not eliminating) inflammation.

**Figure 2:**
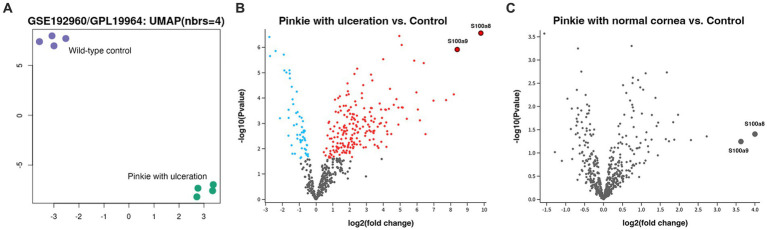
S100a8 and S100a9, known DAMPs, are significantly induced in a mouse model of DED with corneal ulceration compared to the wild-type control with normal corneal epithelium. GEO dataset GSE192960 (platform GPL19964) deposited by Pflugfelder and colleagues was analyzed for differentially expressed genes, with significance taken at an adjusted *p* value of ≤0.05. Panel **(A)** illustrates a uniform manifold approximation and projection (UMAP) clustering analysis, showing the distinct expression patterns in the wild-type control versus the Pinkie DED model with corneal ulceration. Panel **(B)** shows a Volcano plot of differentially expressed genes (DEGs) with the red dots indicating significant up-regulation and the blue dots representing significant down-regulation (gray dots indicate no statistically significant difference). The most highly differentially expressed gene is S100a8, with S100a9 also significantly elevated, as indicated in the figure. Panel **(C)** is a comparison of gene expression between the wild-type control and the Pinkie DED model without ulceration. Note that two of the most highly DEGs in this DED model with corneal ulceration are S100a8 and S100a9, which are also upregulated in the DED model with normal corneas, although the values did not achieve statistical significance.

In support of this idea, in additional experiments, we have shown that DOPG inhibits TLR activation in response to a synthetic lipopeptide TLR2 agonist in corneal epithelial cells (44), as well as to DAMPs known to be elevated with corneal wounding [e.g., (59, 60)], heat shock protein B4 (also known as crystallin alpha A) and S100A proteins in a macrophage cell line and TLR2 and TLR4 reporter cell lines (44, 45). To determine if any of these DAMPs could potentially be involved in DED, we analyzed a dataset available in the Gene Expression Omnibus (GEO) database using GEO2R. These data were obtained by Alam et al. (61) from a DED model, the “Pinkie” mouse strain with a loss-of-function retinoid X receptor (RXRα) mutation, and deposited into GEO. Pinkie mice exhibit characteristic signs of DED, including reduced tear volume, disruption of the corneal barrier, corneal and conjunctival cornification, and Goblet cell loss (61). Over time, these mice can also develop corneal vascularization, opacification, and ulceration (61). We analyzed gene expression in the Pinkie mice with corneal ulcers versus the matched wild-type strain with normal corneas using the GSE192960 dataset (platform GPL19964). UMAP analysis demonstrated a distinct separation of the gene expression profiles between the two groups (Figure 2A). A total of 258 significantly differentially expressed genes were observed between Pinkie mice with corneal ulceration compared to wild-type controls with normal corneas, with S100a8 being the most significantly upregulated gene; S100a9 was also highly and significantly upregulated (Figure 2B). Interestingly, Pinkie mice with normal corneas had no significantly differentially expressed genes compared to control mice (Figure 2C); however, S100a8 and S100a9 tended to be upregulated in these mice as well, although the adjusted *p*-value did not attain statistical significance. Since previously we showed that S100A9 potently activates TLR2 and TLR4, and this activation is inhibited by DOPG (44, 45), these results suggest the possible involvement of S100A8 and S100A9 in DED. Since DOPG inhibits TLR/innate immune system activation and inflammatory mediator expression in various cells (including reporter cell lines expressing the human TLR2 and TLR4 signaling pathway) in response to S100A9 (45), these data also suggest that DOPG may be a possible therapeutic to treat the corneal inflammation observed in DED.

Finally, it should be noted that phosphatidylglycerol is already used in some ophthalmic products. For example, phosphatidylglycerol is used as an intravenous drug delivery agent for the Food and Drug Administration-approved drug Visudyne^®^ (verteporfin), used for photodynamic therapy of age-related macular degeneration and abnormal blood vessels in the eye (62). Similarly, one species of phosphatidylglycerol, dimyristoylphosphatidylglycerol (DMPG) is listed as an inactive ingredient in Systane® Complete eyedrops [containing also propylene glycol, hydroxypropyl guar, mineral oil, polyoxyl 40 stearate, sorbitan tristearate, boric acid, sorbitol, edetate disodium, POLYQUAD* (polidronium chloride) 0.001% preservative, and purified water] (63). However, since Fowler et al. (44) have reported that DMPG can inhibit TLR2 activation by HSPB4 with comparable efficacy to DOPG, DMPG may actually function as an active component to suppress inflammation. Finally, phosphatidylglycerol is being tested for its ability to stabilize the tear film in contact lens wearers (Clinical Trial: ACTRN12613001323718) (64). The use of different forms of phosphatidylglycerol in these various ophthalmic preparations indicates its safety profile, which together with the molecule’s anti-inflammatory properties, would suggest that phosphatidylglycerol, like glycerol, may also have potential utility for the treatment of DED.

### 1.7 Possible advantages of glycerol-based treatments

#### 1.7.1 Wide availability and low cost

Glycerol is a cost-effective and widely available ingredient, making it accessible to many DED patients. Likewise, its non-toxic nature allows frequent use without the risk of adverse effects, especially when added to preservative-free formulations.

### 1.8 Multifunctionality

Unlike artificial tears that provide only moisture and lubrication, glycerol offers not only hydration and lubrication but also osmoprotection, making it a more versatile treatment option. This multiple functionality can address both the symptoms of dryness and the underlying osmotic stress contributing to DED progression. Indeed, as mentioned above, the inclusion of glycerol in artificial tears containing CMC accelerated the onset of sign and symptom relief, even though there was no difference detected with more chronic therapy (32). In addition, as discussed above, glycerol is a precursor for the synthesis of lipids, which serve to stabilize the tear film and are often decreased in the tear film of patients with DED (24). Finally, glycerol also has anti-inflammatory properties, apparently both itself and following its conversion to anti-inflammatory phosphatidylglycerol, as discussed above.

#### 1.8.1 Suitability for sensitive eyes

Multiple patients with DED are sensitive to preservatives used in artificial tears. Glycerol-based artificial tears, commonly known as preservative-free formulations, are safe for patients with sensitive eyes or who require long-term, frequent application. Brands that contain glycerol as their active ingredient include Oasis^®^ and Soothe^®^ (4).

## 2 Discussion and conclusions

While glycerol shows promising results as a treatment for DED, further studies are needed to fully illuminate its anti-inflammatory properties and potential for long-term benefits. Furthermore, research investigating combination therapies containing glycerol and other agents, such as hyaluronic acid, anti-inflammatory drugs or perhaps phosphatidylglycerol, may also yield improved outcomes for DED patients.

Glycerol offers significant therapeutic benefits for patients with DED, primarily through its humectant, osmoprotective, demulcent, and tear film-stabilizing properties. Its efficacy in alleviating DED symptoms, combined with its safety and cost-effectiveness, makes it a valuable option in the current landscape of dry eye treatments. Future studies may further expand its role, particularly in combination with other therapeutic agents, offering promise for more comprehensive management strategies for DED.

## Data Availability

Publicly available datasets were analyzed in this study. These data can be found here: https://www.ncbi.nlm.nih.gov/geo/query/acc.cgi?acc=GSE192960.

## References

[1] Shimazaki J. Definition and diagnostic criteria of dry eye disease: historical overview and future directions. Invest Ophthalmol Vis Sci. (2018) 59:DES7. doi: 10.1167/iovs.17-23475, PMID: 30481800

[2] Matsuda Y Machida M Nakagami Y Nakajima T Azuma M. NFE2L2 activator RS9 protects against corneal epithelial cell damage in dry eye models. PLoS One. (2020) 15:e0229421. doi: 10.1371/journal.pone.0229421, PMID: 32320433 PMC7176120

[3] Roth H Conway T Hollander D. Evaluation of carboxymethylcellulose 0.5%/glycerin 0.9% and sodium hyaluronate 0.18% artificial tears in patients with mild to moderate dry eye. Clin Optomet. (2011) 3:73–8. doi: 10.2147/OPTO.S25571, PMID: 39723116

[4] Kathuria A Shamloo K Jhanji V Sharma A. Categorization of marketed artificial tear formulations based on their ingredients: a rational approach for their use. J Clin Med. (2021) 10:1289. doi: 10.3390/jcm10061289, PMID: 33800965 PMC8003881

[5] Stapleton F Alves M Bunya VY Jalbert I Lekhanont K Malet F. TFOS DEWS II epidemiology report. Ocul Surf. (2017) 15:334–65. doi: 10.1016/j.jtos.2017.05.003, PMID: 28736337

[6] Kuo YK Lin IC Chien LN Lin TY How YT Chen KH. Dry eye disease: a review of epidemiology in Taiwan, and its clinical treatment and merits. J Clin Med. (2019) 8:1227. doi: 10.3390/jcm8081227, PMID: 31443274 PMC6722537

[7] Yagci A Gurdal C. The role and treatment of inflammation in dry eye disease. Int Ophthalmol. (2014) 34:1291–301. doi: 10.1007/s10792-014-9969-x, PMID: 25416345

[8] Recchioni A Mocciardini E Ponzini E Tavazzi S. Viscoelastic properties of the human tear film. Exp Eye Res. (2022) 219:109083. doi: 10.1016/j.exer.2022.109083, PMID: 35460666

[9] Seen S Tong L. Dry eye disease and oxidative stress. Acta Ophthalmol. (2018) 96:e412–20. doi: 10.1111/aos.13526, PMID: 28834388

[10] Galor A Feuer W Lee DJ Florez H Venincasa VD Perez VL. Ocular surface parameters in older male veterans. Invest Ophthalmol Vis Sci. (2013) 54:1426–33. doi: 10.1167/iovs.12-10819, PMID: 23385801 PMC3597187

[11] Modi YS Qurban Q Zlotcavitch L Echeverri RJ Feuer W Florez H. Ocular surface symptoms in veterans returning from operation Iraqi freedom and operation enduring freedom. Invest Ophthalmol Vis Sci. (2014) 55:650–3. doi: 10.1167/iovs.13-13330, PMID: 24408975 PMC3912909

[12] Pouyeh B Viteri E Feuer W Lee DJ Florez H Fabian JA. Impact of ocular surface symptoms on quality of life in a United States veterans affairs population. Am J Ophthalmol. (2012) 153:1061–1066.e3. doi: 10.1016/j.ajo.2011.11.030, PMID: 22330309

[13] Dahal M Kaiti R Nepal S Shyangbo R Dahal G Ghimire R. Dry eye disease: a comprehensive review In: Journal of International Conference Proceedings, vol. 2 (2020) Available at: https://crimsonpublishers.com/icp/fulltext/ICP.000537.php

[14] Feroze KB Zeppieri M Khazaeni L. Steroid-induced Glaucoma. Treasure Island (FL): StatPearls (2024). 28613653

[15] Chu L Wang C Zhou H. Inflammation mechanism and anti-inflammatory therapy of dry eye. Front Med (Lausanne). (2024) 11:1307682. doi: 10.3389/fmed.2024.1307682, PMID: 38420354 PMC10899709

[16] Lemp M Geerling G. Distinguishing evaporative form aqueous deficient dry eye. Cataract & Refractive Surgery Today. Europe. (2011):40–2.

[17] Bustamante-Arias A Ruiz Lozano RE Rodriguez-Garcia A. Dry eye disease, a prominent manifestation of systemic autoimmune disorders. Eur J Ophthalmol. (2022) 32:3142–62. doi: 10.1177/11206721221088259, PMID: 35300528

[18] EK Apkek. (2025). Available at: https://www.hopkinsmedicine.org/health/conditions-and-diseases/dry-eye (Accessed November 18, 2024).

[19] Golden MI Meyer JJ Zeppieri M Patel BC. Dry eye syndrome. Treasure Island (FL): StatPearls (2024). 29262012

[20] Rao SK Mohan R Gokhale N Matalia H Mehta P. Inflammation and dry eye disease-where are we? Int J Ophthalmol. (2022) 15:820–7. doi: 10.18240/ijo.2022.05.20, PMID: 35601175 PMC9091897

[21] Megha KB Joseph X Akhil V Mohanan PV. Cascade of immune mechanism and consequences of inflammatory disorders. Phytomedicine. (2021) 91:153712. doi: 10.1016/j.phymed.2021.153712, PMID: 34511264 PMC8373857

[22] Garcia JI Garcia-Marin H. Glycerol based solvents: synthesis, properties and applications. Green Chem. (2014) 16:1007–33. doi: 10.1039/C3GC41857J

[23] Lanier OL Manfre MG Bailey C Liu Z Sparks Z Kulkarni S. Review of approaches for increasing ophthalmic bioavailability for eye drop formulations. AAPS Pharm Sci Tech. (2021) 22:107. doi: 10.1208/s12249-021-01977-0, PMID: 33719019

[24] Rolando M Merayo-Lloves J. Management strategies for evaporative dry eye disease and future perspective. Curr Eye Res. (2022) 47:813–23. doi: 10.1080/02713683.2022.2039205, PMID: 35521685

[25] Horton M Horton M Reinhard E. Master the maze of artificial tears. Rev Optometry (2018). Available at: https://www.reviewofoptometry.com/article/master-the-maze-of-artificial-tears (Accessed December 19, 2024).

[26] Larson L. (2016). Available at: https://webeye.ophth.uiowa.edu/eyeforum/tutorials/artificial-tears.htm#gsc.tab=0 (Accessed December 19, 2024).

[27] Gensheimer WG Kleinman DM Gonzalez MO Sobti D Cooper ER Smits G. Novel formulation of glycerin 1% artificial tears extends tear film break-up time compared with Systane lubricant eye drops. J Ocul Pharmacol Ther. (2012) 28:473–8. doi: 10.1089/jop.2011.0053, PMID: 22554205

[28] Paugh JR Tse J Nguyen T Sasai A Chen E De Jesus MT. Efficacy of the fluorescein tear breakup time test in dry eye. Cornea. (2020) 39:92–8. doi: 10.1097/ICO.0000000000002148, PMID: 31513046 PMC6893123

[29] Lidich N Garti-Levy S Aserin A Garti N. Potentiality of microemulsion systems in treatment of ophthalmic disorders: keratoconus and dry eye syndrome - *in vivo* study. Colloids Surf B Biointerfaces. (2019) 173:226–32. doi: 10.1016/j.colsurfb.2018.09.063, PMID: 30300828

[30] Kiss HJ Nemeth J. Isotonic glycerol and sodium hyaluronate containing artificial tear decreases Conjunctivochalasis after one and three months: a self-controlled, unmasked study. PLoS One. (2015) 10:e0132656. doi: 10.1371/journal.pone.0132656, PMID: 26172053 PMC4501551

[31] Yvon C Patel BC Malhotra R. Conjunctivochalasis. Treasure Island, FL: StatPearls (2024).

[32] Gupta P Goyal E Gupta D. Evaluating the role of glycerin as an adjuvant in tear substitute eye drops in patients with dry eye. Indian J Basic App Med Res. (2020) 9:167–70. doi: 10.36848/IJBAMR/2020/12210.51270

[33] Lievens C Berdy G Douglass D Montaquila S Lin H Simmons P. Evaluation of an enhanced viscosity artificial tear for moderate to severe dry eye disease: a multicenter, double-masked, randomized 30-day study. Cont Lens Anterior Eye. (2019) 42:443–9. doi: 10.1016/j.clae.2018.12.003, PMID: 30573298

[34] Kiran M Mane A Banerjee B Mehta H Yadav P. A comparative study to evaluate the efficacy of carboxy methyl cellulose with glycerin and balanced electrolytes as excipients vs plain carboxy methyl cellulose, for keeping the eye moist. J Med Sci Clin Res. (2017) 5:18316–22. doi: 10.18535/jmscr/v5i3.09, PMID: 35979512

[35] Wei Y Asbell PA. The core mechanism of dry eye disease is inflammation. Eye Contact Lens. (2014) 40:248–56. doi: 10.1097/ICL.0000000000000042, PMID: 25390549 PMC4231828

[36] Hessen M Akpek EK. Dry eye: an inflammatory ocular disease. J Ophthalmic Vis Res. (2014) 9:240–50. PMID: 25279127 PMC4181208

[37] Hara-Chikuma M Satooka H Watanabe S Honda T Miyachi Y Watanabe T. Aquaporin-3-mediated hydrogen peroxide transport is required for NF-kappaB signalling in keratinocytes and development of psoriasis. Nat Commun. (2015) 6:7454. doi: 10.1038/ncomms8454, PMID: 26100668 PMC5628617

[38] Miller EW Dickinson BC Chang CJ. Aquaporin-3 mediates hydrogen peroxide uptake to regulate downstream intracellular signaling. Proc Natl Acad Sci USA. (2010) 107:15681–6. doi: 10.1073/pnas.1005776107, PMID: 20724658 PMC2936599

[39] Choudhary V Kaddour-Djebbar I Custer VE Uaratanawong R Chen X Cohen E. Glycerol improves skin lesion development in the Imiquimod mouse model of psoriasis: experimental confirmation of anecdotal reports from patients with psoriasis. Int J Mol Sci. (2021) 22:8749. doi: 10.3390/ijms22168749, PMID: 34445455 PMC8395744

[40] Zheng X Bollag WB. Aquaporin 3 colocates with phospholipase D2 in caveolin-rich membrane microdomains and is regulated by keratinocyte differentiation. J Invest Dermatol. (2003) 121:1487–95. doi: 10.1111/j.1523-1747.2003.12614.x, PMID: 14675200

[41] Zheng X Ray S Bollag WB. Modulation of phospholipase D-mediated phosphatidylglycerol formation by differentiating agents in primary mouse epidermal keratinocytes. Biochim Biophys Acta. (2003) 1643:25–36. doi: 10.1016/j.bbamcr.2003.08.006, PMID: 14654225

[42] Bollag WB Xie D Zheng X Zhong X. A potential role for the phospholipase D2-aquaporin-3 signaling module in early keratinocyte differentiation: production of a phosphatidylglycerol signaling lipid. J Invest Dermatol. (2007) 127:2823–31. doi: 10.1038/sj.jid.5700921, PMID: 17597824

[43] Choudhary V Griffith S Chen X Bollag WB. Pathogen-associated molecular pattern-induced TLR2 and TLR4 activation increases keratinocyte production of inflammatory mediators and is inhibited by Phosphatidylglycerol. Mol Pharmacol. (2020) 97:324–35. doi: 10.1124/mol.119.118166, PMID: 32173651 PMC7174787

[44] Fowler TE Choudhary V Melnyk S Farsi M Chang LY Fortingo N. Dioleoylphosphatidylglycerol inhibits heat shock protein B4 (HSPB4)-induced inflammatory pathways in vitro. Int J Mol Sci. (2023) 24:5839. doi: 10.3390/ijms24065839, PMID: 36982926 PMC10059050

[45] Choudhary V Uaratanawong R Patel RR Patel H Bao W Hartney B. Phosphatidylglycerol inhibits toll-like receptor-mediated inflammation by danger-associated molecular patterns. J Invest Dermatol. (2019) 139:868–77. doi: 10.1016/j.jid.2018.10.021, PMID: 30391260 PMC7309510

[46] Chiba H Piboonpocanun S Mitsuzawa H Kuronuma K Murphy RC Voelker DR. Pulmonary surfactant proteins and lipids as modulators of inflammation and innate immunity. Respirology. (2006) 11:S2–6. doi: 10.1111/j.1440-1843.2006.00797.x, PMID: 16423264

[47] Kandasamy P Numata M Berry KZ Fickes R Leslie CC Murphy RC. Structural analogs of pulmonary surfactant phosphatidylglycerol inhibit toll-like receptor 2 and 4 signaling. J Lipid Res. (2016) 57:993–1005. doi: 10.1194/jlr.M065201, PMID: 27095543 PMC4878184

[48] Kandasamy P Zarini S Chan ED Leslie CC Murphy RC Voelker DR. Pulmonary surfactant phosphatidylglycerol inhibits *Mycoplasma pneumoniae*-stimulated eicosanoid production from human and mouse macrophages. J Biol Chem. (2011) 286:7841–53. doi: 10.1074/jbc.M110.170241, PMID: 21205826 PMC3048671

[49] Kuronuma K Mitsuzawa H Takeda K Nishitani C Chan ED Kuroki Y. Anionic pulmonary surfactant phospholipids inhibit inflammatory responses from alveolar macrophages and U937 cells by binding the lipopolysaccharide-interacting proteins CD14 and MD-2. J Biol Chem. (2009) 284:25488–500. doi: 10.1074/jbc.M109.040832, PMID: 19584052 PMC2757950

[50] Numata M Chu HW Dakhama A Voelker DR. Pulmonary surfactant phosphatidylglycerol inhibits respiratory syncytial virus-induced inflammation and infection. Proc Natl Acad Sci USA. (2010) 107:320–5. doi: 10.1073/pnas.0909361107, PMID: 20080799 PMC2806703

[51] Numata M Kandasamy P Nagashima Y Posey J Hartshorn K Woodland D. Phosphatidylglycerol suppresses influenza a virus infection. Am J Respir Cell Mol Biol. (2012) 46:479–87. doi: 10.1165/rcmb.2011-0194OC, PMID: 22052877 PMC3359948

[52] Numata M Mitchell JR Tipper JL Brand JD Trombley JE Nagashima Y. Pulmonary surfactant lipids inhibit infections with the pandemic H1N1 influenza virus in several animal models. J Biol Chem. (2020) 295:1704–15. doi: 10.1074/jbc.RA119.012053, PMID: 31882535 PMC7008372

[53] Numata M Nagashima Y Moore ML Berry KZ Chan M Kandasamy P. Phosphatidylglycerol provides short-term prophylaxis against respiratory syncytial virus infection. J Lipid Res. (2013) 54:2133–43. doi: 10.1194/jlr.M037077, PMID: 23749985 PMC3708363

[54] Osman C Voelker DR Langer T. Making heads or tails of phospholipids in mitochondria. J Cell Biol. (2011) 192:7–16. doi: 10.1083/jcb.201006159, PMID: 21220505 PMC3019561

[55] Voelker DR Numata M. Phospholipid regulation of innate immunity and respiratory viral infection. J Biol Chem. (2019) 294:4282–9. doi: 10.1074/jbc.AW118.003229, PMID: 30733339 PMC6433062

[56] Klein ME Mauch S Rieckmann M Martinez DG Hause G Noutsias M. Phosphatidylserine (PS) and phosphatidylglycerol (PG) nanodispersions as potential anti-inflammatory therapeutics: comparison of *in vitro* activity and impact of pegylation. Nanomedicine. (2020) 23:102096. doi: 10.1016/j.nano.2019.102096, PMID: 31669855

[57] Xie D Choudhary V Seremwe M Edwards JG Wang A Emmons AC. Soy Phosphatidylglycerol reduces inflammation in a contact irritant ear edema mouse model in vivo. J Pharmacol Exp Ther. (2018) 366:1–8. doi: 10.1124/jpet.117.244756, PMID: 29695409 PMC5988020

[58] Bollag WB Olala LO Xie D Lu X Qin H Choudhary V. Dioleoylphosphatidylglycerol accelerates corneal epithelial wound healing. Invest Ophthalmol Vis Sci. (2020) 61:29. doi: 10.1167/iovs.61.3.29, PMID: 32186673 PMC7401755

[59] Bettahi I Sun H Gao N Wang F Mi X Chen W. Genome-wide transcriptional analysis of differentially expressed genes in diabetic, healing corneal epithelial cells: hyperglycemia-suppressed TGFbeta3 expression contributes to the delay of epithelial wound healing in diabetic corneas. Diabetes. (2014) 63:715–27. doi: 10.2337/db13-1260, PMID: 24306208 PMC3900551

[60] Oh JY Choi H Lee RH Roddy GW Ylostalo JH Wawrousek E. Identification of the HSPB4/TLR2/NF-kappaB axis in macrophage as a therapeutic target for sterile inflammation of the cornea. EMBO Mol Med. (2012) 4:435–48. doi: 10.1002/emmm.201200221, PMID: 22359280 PMC3403300

[61] Alam J Yazdanpanah G Ratnapriya R Borcherding N de Paiva CS Li D. IL-17 producing lymphocytes cause dry eye and corneal disease with aging in RXRalpha mutant mouse. Front Med (Lausanne). (2022) 9:849990. doi: 10.3389/fmed.2022.849990, PMID: 35402439 PMC8983848

[62] Available at: https://www.ema.europa.eu/en/documents/product-information/visudyne-epar-product-information_en.pdf (Accessed November 18, 2024).

[63] Available at: https://systane-ca.myalcon.com/ca-en/eye-care/systane/products/systane-complete/ingredients/#:~:text=SYSTANE%C2%AE%20COMPLETE%20Lubricant%20Eye%20Drops%20is%20a%20sterile%20translucent,)%200.00125%20preservative%2C%20and%20purified (Accessed November 18, 2024).

[64] Rohit A Willcox M Mitchell TW Stapleton F. Effect of a lipid emulsion drop on tear film characteristics of habitual contact lens wearers. Invest Ophthalmol Vis Sci. (2015) 56:3156A.

